# Epidemiology of Antepartum Stillbirths in Austria—A Population-Based Study between 2008 and 2020

**DOI:** 10.3390/jcm10245828

**Published:** 2021-12-13

**Authors:** Dana Anaïs Muin, Hanns Helmer, Hermann Leitner, Sabrina Neururer

**Affiliations:** 1 Department of Obstetrics and Gynecology, Division of Fetomaternal Medicine, Comprehensive Center for Pediatrics, Medical University of Vienna, Waehringer Guertel 18-20, 1090 Vienna, Austria; hanns.helmer@meduniwien.ac.at; 2 Department of Clinical Epidemiology, Tyrolean Federal Institute for Integrated Care, Tirol Kliniken GmbH, Anichstraße 35, 6020 Innsbruck, Austria; hermann.leitner@tirol-kliniken.at (H.L.); s.neururer@tirol-kliniken.at (S.N.)

**Keywords:** stillbirth, fetal death, perinatal mortality, epidemiology, registration

## Abstract

(1) Background: Across Europe, the incidence of antepartum stillbirth varies greatly, partly because of heterogeneous definitions regarding gestational weeks and differences in legislation. With this study, we sought to provide a comprehensive overview on the demographics of antepartum stillbirth in Austria, defined as non-iatrogenic fetal demise ≥22^+0^ gestational weeks (/40). (2) Methods: We conducted a population-based study on epidemiological characteristics of singleton antepartum stillbirth in Austria between January 2008 and December 2020. Data were derived from the validated Austrian Birth Registry. (3) Results: From January 2008 through December 2020, the antepartum stillbirth rate ≥20^+0^/40 was 3.10, ≥22^+0^/40 3.14, and ≥24^+0^/40 2.83 per 1000 births in Austria. The highest incidence was recorded in the federal states of Vienna, Styria, and Lower and Upper Austria, contributing to 71.9% of all stillbirths in the country. In the last decade, significant fluctuations in incidence were noted: from 2011 to 2012, the rate significantly declined from 3.40 to 3.07‰, whilst it significantly increased from 2.76 to 3.49‰ between 2019 and 2020. The median gestational age of antepartum stillbirth in Austria was 33^+0^ (27^+2^–37^+4^) weeks. Stillbirth rates ≤26/40 ranged from 164.98 to 334.18‰, whilst the lowest rates of 0.58–8.4‰ were observed ≥36/40. The main demographic risk factors were maternal obesity and low parity. (4) Conclusions: In Austria, the antepartum stillbirth rate has remained relatively stable at 2.83–3.10 per 1000 births for the last decade, despite a significant decline in 2012 and an increase in 2020.

## 1. Introduction

Live birth rates and perinatal mortality statistics are considered two of the paramount parameters which shape national demographics and reflect the general population qualities and public health standards within a system. Whilst these may differ grossly between low- and high-income countries across the world, basic characteristics in live birth and stillbirth rates are approximately similar within Europe [1]. After all, minor influences in regional legislation and definition may contribute to major differences even within Europe, which make continental stillbirth rates, in particular, range from 2.6 to 9.1 per 1000 total births [2,3].

The differences in the legislation of perinatal mortality statistics primarily concern definitions of stillbirth with regard to gestational age of delivery, birth weight, and timing or way of demise. While some countries differentiate between antepartum and peripartum fetal death, others include terminations of pregnancies into their fetal mortality statistics [4]. Since the Lancet Stillbirth Series in 2011, a common consciousness emerged for the necessity to harmonize definitions, reporting standards, and legislations internationally to allow valid comparisons between nations and expand knowledge for better prevention of stillbirths worldwide [5].

In Austria, stillbirth is defined as the delivery of a baby of ≥500 g birth weight, irrespective of gestational age, with no signs of life, such as pulsation of the umbilical cord, positive heartbeats, and involuntary muscle contractions. Perinatal mortality is defined as the summary of both stillbirth and early neonatal death up to seven days of life. The Austrian perinatal mortality statistics are annually published by Statistics Austria and gather live birth and mortality data from the Austrian Birth Registry. 

To date, stillbirth data as such are not precisely differentiated between the actual timing of stillbirth (i.e., antepartum versus intrapartum) and termination of pregnancy. In view of the profound differences in both the etiology and clinical implications of antepartum versus intrapartum stillbirths, we hereby aim to provide accurate and clean data from the Austrian Birth Registry to portray the demography of antepartum stillbirths in Austria since the implementation of the registry. We furthermore set out to assess regional differences in antepartum stillbirths and evaluate potential risk factors in the Austrian population.

## 2. Materials and Methods

### 2.1. Data Collection

The Austrian Birth Registry, founded in 2008, is maintained by the Institute of Clinical Epidemiology, Tyrolean Federal Institute for Integrated Care (https://www.iet.at/) and collects perinatal data from all maternity units in Austria (Supplementary Figure S1). It provides epidemiological data from all deliveries for Austria’s Federal Statistical Office (https://pic.statistik.at). Data are regularly checked for accuracy and consistency and therefore assure validity.

**Figure 1:**
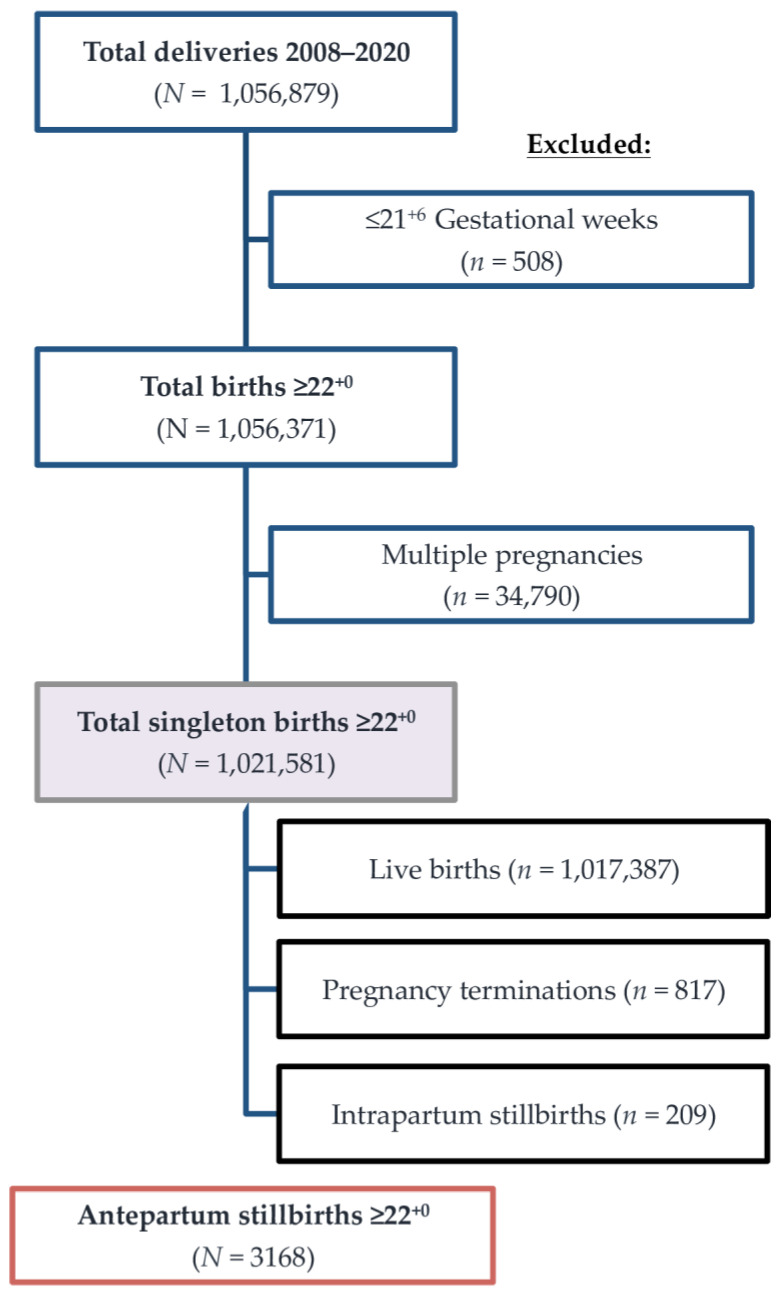
Flowchart on eligibility and selection of study population.

For this study and statistical analyses, data from the Austrian Birth Registry were extracted which fulfilled the following criteria: singleton intrauterine fetal death (IUFD) above 22 weeks of gestation followed by stillbirth between 1 January 2008 and 31 December 2020 at an Austrian maternity unit. Exclusion criteria were terminations of pregnancy, and intrapartum or perinatal demise (Figure 1). For the sake of international comparison, the incidence of IUFDs above 20 weeks of gestation, excluding terminations, is provided.

### 2.2. Statistical Analyses

Continuous data are described as mean (M) and standard deviation (SD) or median and 25th and 75th percentiles (interquartile range, IQR). Categorical data are described as absolute (n) and relative frequencies (%). Comparison of categorical variables was conducted with the chi^2^ (_X_^2^) test; comparison of continuous data was conducted with an independent t-test and the Wilcoxon rank test. Discrepancy between values was reported with standard error of the mean (SEM) and the value of discrepancy ± standard deviation of discrepancy with a 95% confidence interval (CI). A binary logistic regression was performed in order to assess the influence of feto-maternal characteristics on stillbirth. A two-tailed *p*-value below 0.05 was considered significant. Statistical tests were performed with GraphPad Prism 9 for macOS (GraphPad Software, LLC) and STATA (16.0, StataCorp LLC, College Station, TX, USA). Figures were designed with GraphPad Prism 9 and Microsoft Excel (Version 16.53; Microsoft Corporation, Redmond, WA, USA).

### 2.3. Ethical Approval and Consent

This study was approved by the Ethics Committee of the Medical University of Vienna (Registration number 1154/2019) and complied with the principles as outlined in the Declaration of Helsinki and Good Clinical Practice guidelines. Participants’ written consent was not required as per the Austrian Federal Act (Protection of Personal Data Regulation, §46, Paragraph 1; 2000).

## 3. Results

### 3.1. Incidence of Antepartum Stillbirths in Austria

**Figure 2:**
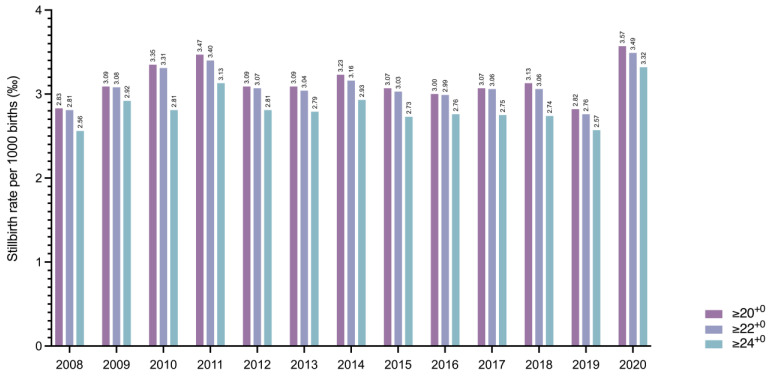
Interleaved contingency bar graph showing the stillbirth rate per 1000 births ≥20^+0^, ≥22^+0^, and ≥24^+0^ gestational weeks in Austria between 2008 and 2020.

From January 2008 through December 2020, a total of 2888 antepartum stillbirths were registered ≥24^+0^ gestational weeks in Austria, resulting in a national stillbirth rate of 2.83 ± 0.21 per 1000 births. After lowering the threshold of fetal death to ≥20^+0^ and ≥22^+0^ gestational weeks, a total of 3208 and 3168 fetal deaths, respectively, occurred, increasing the rate to 3.14 ± 0.22 and 3.10 ± 0.21 per 1000 births, respectively (Figure 2).

In total, 275 (8.69%) antepartum singleton stillbirths were registered between 22^+0^ and 23^+6^ gestational weeks, 573 (18.12%) stillbirths occurred between 24^+0^ and 27^+6^, and 2315 (73.19%) stillbirths occurred ≥28^+0^ gestational weeks in Austria from 2008 to 2020.

**Figure 3:**
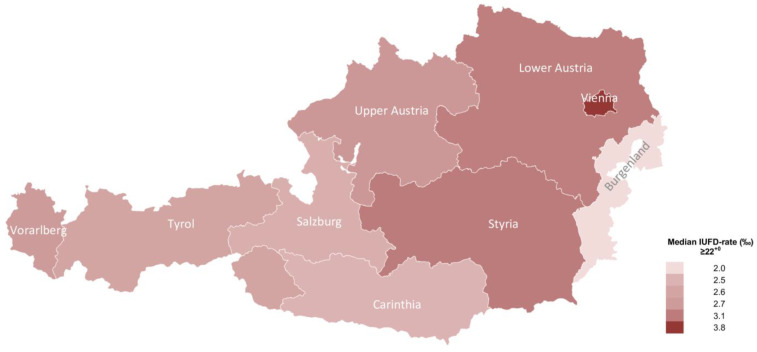
Map chart illustrating the rate of antepartum stillbirths per 1000 births ≥22^+0^ weeks of gestation in the nine Austrian federal states between January 2008 and December 2020.

Across the country, the highest incidence of antepartum stillbirth ≥22^+0^ was registered in the capital state, Vienna, with a rate of 3.81 ± 0.33 per 1000 births, contributing to 28.0% (*n* = 884) of all stillbirths in Austria. The lowest rate was reported in the federal state of Burgenland, with an incidence of 2.07 ± 0.74 antepartum stillbirths per 1000 births (Figure 3). A total of 71.9% (*n* = 2274) of all stillbirths in Austria occurred in the states Lower Austria (*n* = 499; 15.78%), Upper Austria (*n* = 489; 15.46%), Styria (*n* = 402; 12.71%), and Vienna between 2008 and 2020.

Maternal and fetal characteristics per federal state are shown in Table 1. While the antepartum stillbirth rate was the highest in Vienna, demographics show no significant difference regarding maternal age or BMI compared to other states in Austria; however, smoking has been more commonly registered among Viennese women.

**Figure 4:**
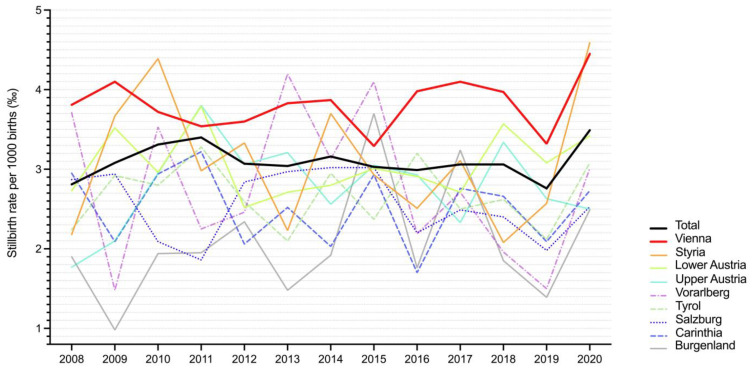
Trends in rates of antepartum stillbirth ≥22^+0^ gestational weeks in Austria between January 2008 and December 2020.

Longitudinal analysis on the trend of the incidence over time showed a significant change and increase in all states across Austria (Figure 4). The highest rate discrepancy over time was reported in Vorarlberg (SEM 0.25; 2.79 ± 0.92 (95% CI 2.23–3.34); *p* < 0.0001), followed by Styria (SEM 0.23; 3.1 ± 0.81 (95% CI 2.61–3.59); *p* < 0.0001) and Burgenland (SEM 0.20; 2.07 ± 0.74 (95% CI 1.63–2.52); *p* < 0.0001). The lowest, yet still statistically significant, time trend was observed in Vienna (SEM 0.09; 3.81 ± 0.33 (95% CI 3.62–4.01); *p* < 0.0001).

From 2011 to 2012, the total antepartum stillbirth rate ≥22 gestational weeks significantly declined from 3.40 to 3.07‰, whereas from 2019 to 2020, it significantly increased from 2.76 to 3.49‰. Thus far, the rate of 2.76‰ in the year 2019 has been the lowest rate of antepartum stillbirths ≥22^+0^ ever documented in Austria since the implementation of the Austrian Birth Registry.

### 3.2. Timing of Antepartum Stillbirths 

**Figure 5:**
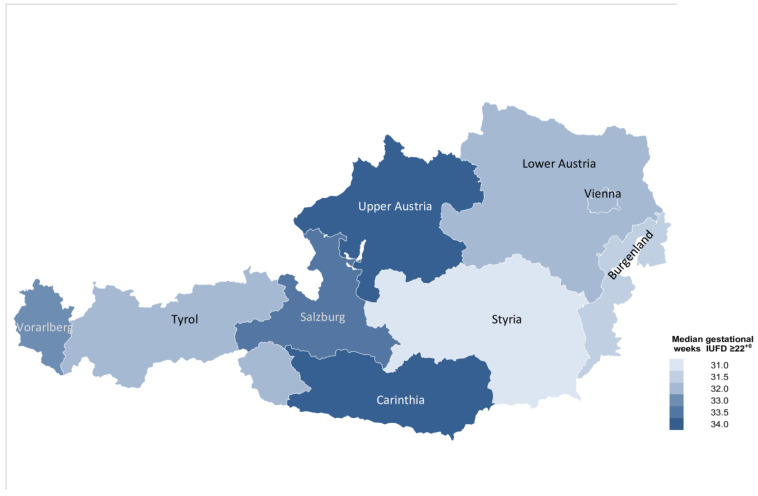
Map chart illustrating the median gestational age of antepartum stillbirths ≥22^+0^ gestational weeks across Austria between 2008 and 2020.

The median gestational age of antepartum stillbirth in Austria between 2008 and 2020 was 33^+0^ (27^+2^–37^+4^) weeks. Stillbirths at higher gestational weeks more frequently occurred in Upper Austria (34^+2^ (29^+1^–38^+1^) weeks), whereas stillbirths in early gestational weeks were more commonly reported in Styria (31^+6^ (26^+1^–36^+6^) weeks) (Figure 5).

**Figure 6:**
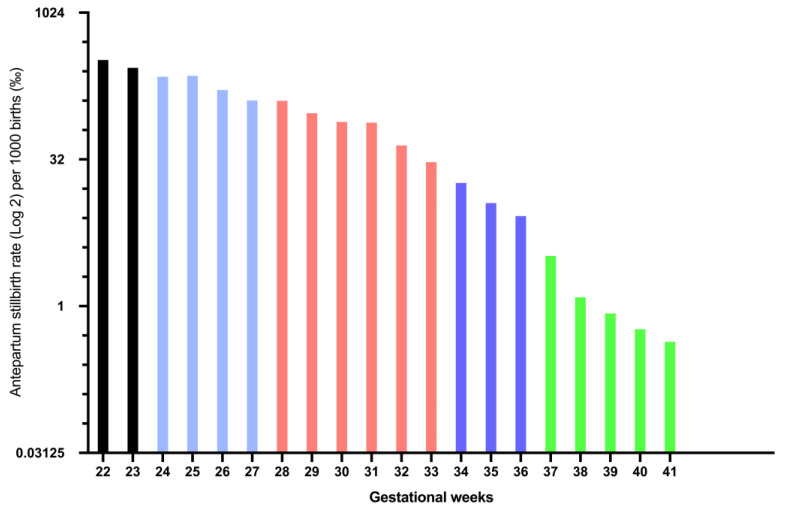
Bar graph illustrating the prevalence of antepartum stillbirth in Austria per gestational week (stillbirth rate per 1000 births in logarithmic scale to the base 2).

Considering the gestational age throughout the study period, the highest prevalence of fetal deaths was noted for pregnancies below 26 gestational weeks, with a rate ranging from 164.98‰ at 26^+0^ to 334.18‰ at 22^+0^. The lowest prevalence was observed above 35 gestational weeks, with a rate ranging from 8.4‰ at 36^+0^ to 0.84‰ at 39^+0^ and 0.58‰ at term at 40^+0^ gestational weeks (Figure 6).

**Figure 7:**
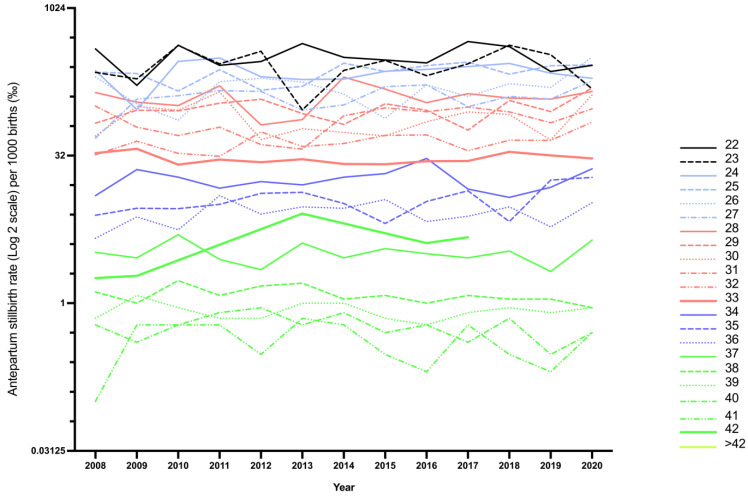
Time trend of stillbirth prevalence as per gestational age in Austria between January 2008 and December 2020: stillbirth rate per 1000 births in logarithmic scale to the base 2.

Over the years, there have been significant fluctuations in the stillbirth prevalence as per age of gestation (Figure 7). The highest fluctuation over time was observed at weeks 22 and 23, with a discrepancy in prevalence of SEM 25.52 (328.8 ± 92.0 (95% CI 273.2–384.4); *p* < 0.0001) and SEM 28.07 (271.9 ± 101.2 (95% CI 210.8–333.1); *p* < 0.0001), respectively. Meanwhile, in 2017, the antepartum stillbirth rate peaked at 466.7‰ for fetuses of 22^+0^–22^+6^ gestational age, and it significantly declined to 230.8‰ in 2019, reflecting a reduction of 235.9‰ within only two years. An even greater reduction of 274.8‰ was noted for stillborn fetuses at 23^+0^–23^+6^ gestation, with a significant decrease from 428.6‰ in 2018 to 153.8‰ in 2020. The steadiest prevalence was observed for stillbirths occurring ≥38 gestational weeks.

### 3.3. Risk Factors for Suffering Antepartum Stillbirth in Austria

Factors associated with antepartum stillbirth in Austria are increased maternal BMI (OR 0.98 (95% CI 0.972–0.989); *p* < 0.001), nulliparity (OR 1.32 (95% CI 1.031–1.679); *p* < 0.001), primiparity (OR 1.04 (95% CI 0.822–1.327); *p* = 0.027), and fetal growth restriction (OR 1.00 (95% CI 1.001–1.001); *p* < 0.001). Maternal age (OR 1.00 (95% CI 0.998–1.015); *p* = 0.148), nicotine consumption (OR 1.14 (95% CI 0.989–1.325); *p* = 0.070), and fetal sex (OR 1.08 (95% CI 0.986–1.188); *p* = 0.096) showed no significant association in logistic regression analyses. 

## 4. Discussion

### 4.1. Main Findings and Interpretation

With this population-based study, we sought to assess the antepartum stillbirth rate in a validated cohort restricted to singleton fetuses excluding terminations of pregnancies and intrapartum or peripartum fetal demise in Austria. We generated four main findings, which are of note. 

First, reflecting the medical advances in neonatal intensive care for extremely premature infants [6,7], our Austrian data confirm a significant and steep reduction in stillbirths for gestational weeks 22^+0^ to 23^+6^, which is regarded as the period of fetal viability. Although data are missing regarding the precise cause of death in these fetuses, we may assume that the reduction in stillbirths at such a gestational age in recent years may have involved fetuses who had been rescued from intrauterine demise by early delivery and thus were subjected to iatrogenic extreme prematurity. 

Second, our temporal analysis over the last 13 years showed two significant changes in the overall incidence of antepartum stillbirths in Austria: whilst the average stillbirth rate has remained relatively stable, there was a significant decline in the national antepartum stillbirth rate between January 2011 and December 2012 by 0.34‰ and a significant increase between January 2019 and December 2020 by 0.73‰. In a previous population-based study, we investigated the effect of the implementation of universal gestational diabetes screening (OGTT) in an Austrian pregnant population within the frame of the Mother and Child Booklet (Muin et al. 2021 Manuscript under revision): in consideration of singleton antepartum stillbirths ≥24^+0^ gestational weeks, we found that, whilst, following the implementation of OGTT between 24^+0^ and 28^+0^ in the year 2011, the annual stillbirth rate in the general population remained stable with 2.76 to 2.74 per 1000 births (*p* = 0.845), the stillbirth rate declined from 4.10 to 2.96 per 1000 live births (*p* = 0.043), resulting in an absolute risk reduction of 0.11% and a relative risk reduction of 27.73% in women at greater risk for stillbirths. Despite the lack of valid data on how many women had indeed received treatment for gestational diabetes, we acknowledge that untreated gestational diabetes may contribute to placental dysfunction, causing intrauterine hypoxia and, furthermore, disturbing the fetal metabolic state, resulting in acidosis, especially in later stages of pregnancy; early detection and, therefore, treatment of maternal diabetes are considered important measures in preventing fetal death in these women. 

The pandemic caused by the coronavirus SARS-CoV-2 (COVID-19) in 2020 has conveyed profound direct and indirect effects on pregnant women worldwide. In a population-based study involving singleton antepartum stillbirths ≥24^+0^ gestational weeks, we confirmed that, during the pandemic in Austria, the national stillbirth rate had increased from 2.49‰ to 2.60‰ (*p* = 0.601), yielding a significant increase during the first lockdown phase (*p* = 0.021), with an adjusted odds ratio of 1.57 (95% CI 1.08–2.27; *p* = 0.018), compared to matched historical months [8]. We, therefore, assume that the significant increase by 0.73‰ from 2019 to 2020, as shown by our present data on stillbirths ≥22^+0^ weeks of gestation, may have been, partly, an effect of the pandemic, as observed elsewhere [9,10,11,12,13,14,15,16,17].

Our third imminent finding is the geographical distribution of antepartum stillbirths being centered in and around the capital city of Vienna, accounting for approximately 50% of all antepartum stillbirth cases in Austria. In a previous study, we illustrated the adverse perinatal outcome by increased light pollution, as naturally observed in urban areas [18]. At the same time, we acknowledge a provider bias in larger hospitals, as supported by more accurate registrations of stillbirths in maternity units, along with a higher prevalence of dealing with high-risk pregnancies. Additionally, urbanization has been consistently associated with poorer lifestyle habits and thus greater health risks, which might, therefore, also account for overall greater perinatal risks [19,20].

Our fourth finding of note is the prevalence of stillbirth as per gestational age: In acknowledgement of varying definitions and legal cut-offs for registering fetal deaths as stillbirths versus late miscarriages, the current study confirms that when counting stillbirths ≥28 weeks, as suggested by the WHO, only 73% of all late stillbirths would be represented in our country, i.e., 27% of losses ≥22 weeks would be neglected in their registration. Additionally, taking the threshold ≥24 weeks, as supposed by a birth weight ≥500 g to officially register a stillbirth as per current Austrian law, approximately 9% of late stillbirths (≥22 weeks) are left underrepresented in annual official statistics. This finding supports prior population-based studies showing the underestimated and unrecognized burden of late fetal losses which are not fully acknowledged by law due to limitations in local legislations [2,3,21].

### 4.2. Strengths and Limitations

The accuracy and validity of the dataset from the Austrian Birth Registry provide a precise overview on the epidemiological landscape in Austria. The exact differentiation between three different thresholds for antepartum stillbirth definition, as early as by gestational week 20^+0^, may allow future accurate comparison with international data. Furthermore, we strictly defined antepartum stillbirth as singleton fetal death in utero, excluding elective termination of pregnancy, multiple pregnancies, and perinatal deaths, as epidemiology and etiology in these are known to differ greatly and may cause heterogeneity in incidence and data interpretation.

Our study is limited by its retrospective, multicenter setting with data unavoidably missing and potential data errors inherent to recall bias in participating maternity units. We also acknowledge the lack of a national classification system for defining the cause of fetal death and are, therefore, unable to provide an overview on causes of stillbirth in Austria, as yet. In consideration of the importance of assessing the cause of fetal death in each case, we are currently establishing a prospective national collaboration for acquisition of post-mortem diagnoses for future public health measures. 

## 5. Conclusions

In Austria, the antepartum stillbirth rate per 1000 births was 3.10 for ≥20^+0^ gestational week, 3.14 for ≥22^+0^ gestational week, and 2.83 for ≥24^+0^ gestational week from 2008 to 2020. Whilst there was a significant decline from 2011 to 2012, followed by a significant increase from 2019 to 2020, the overall rate has remained relatively stable compared to other European countries. The most prevalent risk factors were high maternal BMI and low parity. 

## Figures and Tables

**Table 1:** Maternal and fetal characteristics of antepartum stillbirths ≥22^+0^ gestational weeks in Austria between January 2008 and December 2020.

Antepartum Stillbirth		Total	Non-Specified	Vienna	Styria	Lower Austria	Upper Austria	Tyrol	Salzburg	Carinthia	Vorarlberg	Burgenland	*p*-Value ^4^
Total deliveries (*n*)		1,021,581	32,165	231,674	130,211	163,290	175,254	88,325	66,727	57,734	48,785	27,416	.
Total antepartum stillbirths (*n*; ≥22^+0^)		3163	146	884	402	499	489	236	170	145	135	57	<0.001
Antepartum stillbirth rate (‰; ≥22^+0^)		3.10	.	3.82	3.09	3.06	2.79	2.67	2.55	2.51	2.77	2.08	<0.001
Median gestational age of stillbirth (IQR)		33^+0^ (27^+2^–37^+4^)	32^+3^ (27^+6^–37^+6^)	32^+6^ (26^+5^–37^+2^)	31^+6^ (26^+1^–36^+6^)	32^+0^ (26^+5^–37^+0^)	34^+2^ (29^+1^–38^+1^)	32^+5^ (27^+6^–37^+2^)	33^+5^ (28^+5^–38^+0^)	34^+3^ (27^+3^–38^+3^)	33^+0^ (28^+0^–37^+5^)	32^+1^ (27^+5^–36^+6^)	<0.001
Median maternal age at stillbirth (IQR)		31 (26–35)	32 (29–36)	30 (26–35)	31 (26–35)	31 (27–35)	30 (26–34)	31 (27–35)	31 (27–36)	30 (26–34)	31 (27–36)	33 (29–37)	0.188
Median BMI (IQR; kg/m ^2^)		23.4 (20.7–27.0)	22.9 (20.7–26.9)	23.4 (20.7–27.3)	23.4 (20.4–26.6)	23.8 (21.4–27.1)	23.5 (20.8–27.6)	22.8 (20.7–25.7)	22.8 (20.2–26.9)	23.1 (20.9–26.0)	22.4 (20.3–27.7)	23.5 (21.8–28.0)	0.244
BMI category ^1^	Underweight	122 (6.0%)	3 (5.3%)	38 (6.6%)	16 (6.9%)	6 (2.1%)	24 (7.2%)	17 (7.7%)	9 (6.9%)	3 (2.7%)	4 (6.0%)	2 (5.9%)	0.241
	Normal weight	1205 (59.0%)	36 (63.2%)	330 (57.1%)	138 (59.5%)	166 (59.1%)	182 (54.7%)	140 (63.6%)	76 (58.5%)	76 (67.9%)	41 (61.2%)	20 (58.8%)	
	Overweight	443 (21.7%)	12 (21.1%)	126 (21.8%)	49 (21.1%)	70 (24.9%)	73 (21.9%)	45 (20.5%)	28 (21.5%)	23 (20.5%)	10 (14.9%)	7 (20.6%)	
	Obese	274 (13.4%)	6 (10.5%)	84 (14.5%)	29 (12.5%)	39 (13.9%)	54 (16.2%)	18 (8.2%)	17 (13.1%)	10 (8.9%)	12 (17.9%)	5 (14.7%)	
Parity	0	1605 (50.7%)	90 (61.6%)	447 (50.6%)	191 (47.5%)	251 (50.3%)	244 (49.9%)	119 (50.4%)	94 (55.3%)	80 (55.2%)	62 (45.9%)	27 (47.4%)	0.201
	1–3	1416 (44.8%)	48 (32.9%)	387 (43.8%)	193 (48.0%)	226 (45.3%)	222 (45.4%)	114 (48.3%)	71 (41.8%)	64 (44.1%)	65 (48.1%)	26 (45.6%)	
	≥4	142 (4.5%)	8 (5.5%)	50 (5.7%)	18 (4.5%)	22 (4.4%)	23 (4.7%)	3 (1.3%)	5 (2.9%)	1 (0.7%)	8 (5.9%)	4 (7.0%)	
Nicotine consumption		327 (10.3%)	13 (8.9%)	131 (14.8%)	38 (9.5%)	39 (7.8%)	35 (7.2%)	22 (9.3%)	20 (11.8%)	15 (10.3%)	9 (6.7%)	5 (8.8%)	<0.001
Obstetric risk factors ^2^		200 (6.3%)	2 (1.4%)	58 (6.6%)	59 (14.7%)	30 (6.0%)	16 (3.3%)	18 (7.6%)	0 (0 %)	8 (5.5%)	5 (3.7%)	4 (7.0%)	<0.001
Fetal sex ^3^	Male	1639 (52.0%)	71 (49.0%)	479 (54.3%)	196 (48.9%)	251 (50.8%)	265 (54.4%)	129 (54.7%)	89 (53.0%)	63 (43.4%)	62 (45.9%)	34 (60.7%)	0.096
	Female	1510 (48.0%)	74 (51.0%)	403 (45.7%)	205 (51.1%)	243 (49.2%)	222 (45.6%)	107 (45.3%)	79 (47.0%)	82 (56.6%)	73 (54.1%)	22 (39.3%)	
Median birth weight (g) [IQR]		1700 (860–2700)	1570 (850–2810)	1634.5 (781–2685)	1575 (770–2580)	1590 (817–2643)	1988 (1050–2850)	1565 (868–2625)	1815 (990–2776)	1912 (890–2810)	1634 (930–2740)	1380 (870–2555)	<0.001
Median birth height (cm) [IQR]		43 (35–49)	42 (35–50)	43 (34–49)	42 (34–49)	42 (34–49)	45 (36–50)	42 (35–49)	44 (38–50)	46 (36–51)	43 (36–50)	41.5 (34–48)	<0.001

Note: ^1^ *n* = 1119 missing. BMI category: underweight <18.5 kg/m^2^; normal weight 18.6–24.9 kg/m^2^; overweight 25.0–29.9 kg/m^2^; obese ≥30.0 kg/m^2^. ^2^ Obstetric risk factors including risk for premature delivery and/or adverse neonatal outcome. ^3^ *n* = 14 missing data. ^4^ _X_^2^ test for categorical variables, t-test for continuous variables. Abbreviations: BMI, body mass index; IQR, interquartile range.

## Data Availability

The data that support the findings of this study are available from the corresponding author, D.A.M., upon reasonable request.

## Supplementary Materials

The following are available online at https://www.mdpi.com/article/10.3390/jcm10245828/s1, Figure S1: Maternity units contributing with their perinatal data to the Austrian Stillbirth Registry (as per 1 October 2021).
