# Supplementary figures and images for: Epidemiology of Antepartum Stillbirths in Austria—A Population-Based Study between 2008 and 2020
Source: J Clin Med. 2021 Dec 13;10(24):5828. doi: 10.3390/jcm10245828 (PMC8709287; doi:10.3390/jcm10245828)

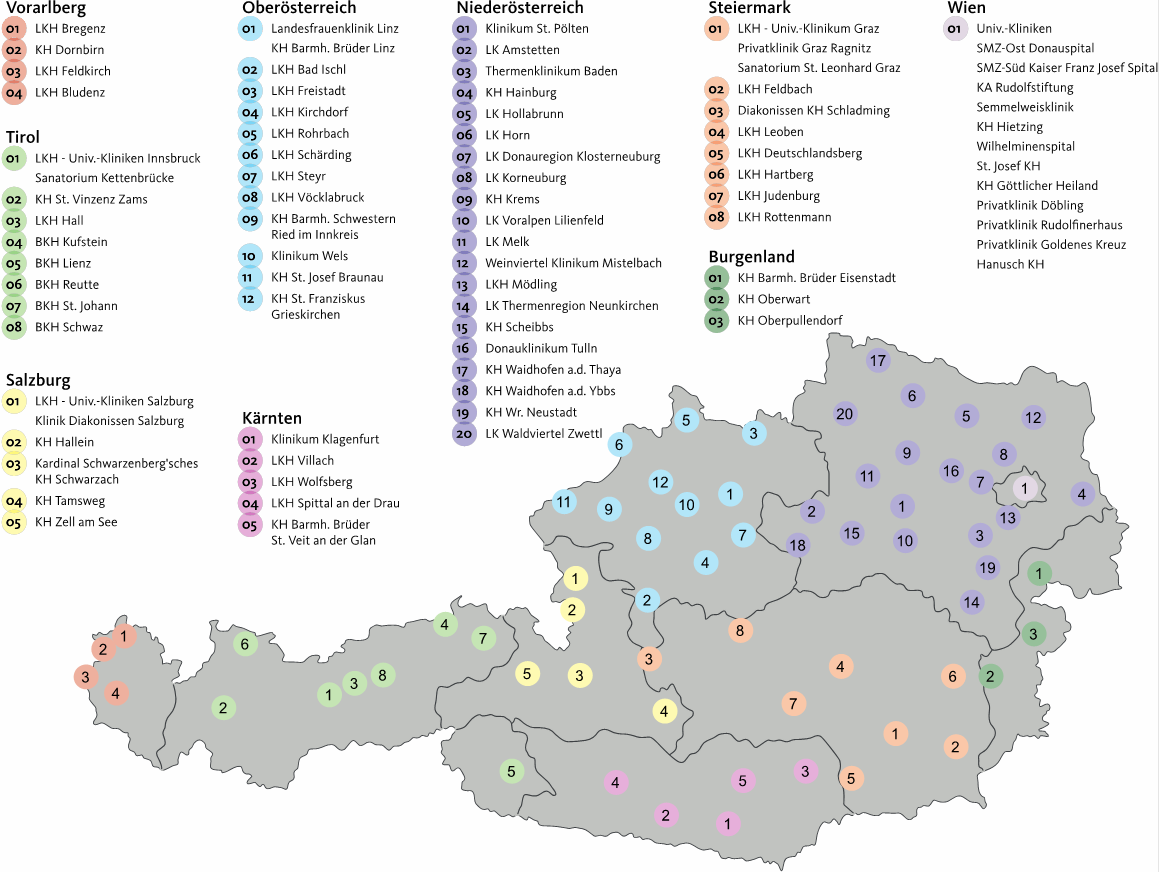

Supplement: Supplementary file 1 [file jcm-10-05828-s001.zip › jcm-1461924-supplementary.png]
